# Clustering via hypergraph modularity

**DOI:** 10.1371/journal.pone.0224307

**Published:** 2019-11-06

**Authors:** Bogumił Kamiński, Valérie Poulin, Paweł Prałat, Przemysław Szufel, François Théberge

**Affiliations:** 1 SGH Warsaw School of Economics, Warsaw, Poland; 2 The Tutte Institute for Mathematics and Computing, Ottawa, ON, Canada; 3 Department of Mathematics, Ryerson University, Toronto, ON, Canada; Universitá degli Studi di Milano, ITALY

## Abstract

Despite the fact that many important problems (including clustering) can be described using hypergraphs, theoretical foundations as well as practical algorithms using hypergraphs are not well developed yet. In this paper, we propose a hypergraph modularity function that generalizes its well established and widely used graph counterpart measure of how clustered a network is. In order to define it properly, we generalize the Chung-Lu model for graphs to hypergraphs. We then provide the theoretical foundations to search for an optimal solution with respect to our hypergraph modularity function. A simple heuristic algorithm is described and applied to a few illustrative examples. We show that using a strict version of our proposed modularity function often leads to a solution where a smaller number of hyperedges get cut as compared to optimizing modularity of 2-section graph of a hypergraph.

## 1 Introduction

An important property of complex networks is their community structure, that is, the organization of vertices in clusters, with many edges joining vertices of the same cluster and comparatively few edges joining vertices of different clusters [1, 2]. In social networks, communities may represent groups by interest (practical application include collaborative tagging—[3]), in citation networks they correspond to related papers (see [4]), similarly in the web communities are formed by pages on related topics.

Yet another example could be financial markets where we have several groups of financial instruments that might be correlated with each other in several different groups. Such groups can be represented as hyperedges and hence detection of communities in such hypergraph could lead to better understanding of dependencies between financial instruments.

Hypergraphs can also be used to model transportation systems. For example in [5] the authors consider transportation system represented as a directed hypergraph. A hyperedge can represent a situation where a single stop (starting or destination point) is being serviced by several public transportation lines and vehicle types that can be differently chosen by an agent traveling within the transportation grid and communities can represent paths taken often together. Another application was presented in [6]—the authors suggest using hypergraphs to model interactions between biological cells in computational biology. Finally, in [7] one can find a discussion on how hypergraphs can be used for modeling telecommunication systems and it is argued that using hypergraphs to represent communication within a mobile grid conveys more information that using regular graphs. Again, this information can be used for a social community detection.

Being able to identify communities in a network could help us to exploit this network more effectively. For example, clusters in citation graphs may help to find similar scientific papers, discovering users with similar interests is important for targeted advertisement, clustering can also be used for network compression and visualization.

The key ingredient for many clustering algorithms is *modularity*, which is at the same time a global criterion to define communities, a quality function of community detection algorithms, and a way to measure the presence of community structure in a network. Modularity for graphs was introduced by Newman and Girvan [8] and it is based on the comparison between the actual density of edges inside a community and the density one would expect to have if the vertices of the graph were attached at random regardless of community structure, while respecting the vertices’ degree on average. This random family of graphs is known as the Chung-Lu random model [9].

Myriad of problems can be described in hypergraph terms, however, despite being formally defined in the 1960s (and various realizations studied long before that) hypergraph theory is patchy and often not sufficiently general. The result is a lack of machinery for investigating hypergraphs, leading researchers and practitioners to create the 2-section graph of a hypergraph of interest [10–16] or to restrict their study to *d*-uniform hypergraphs [17, 18]. In taking the 2-section (that is, making each hyperedge a clique) we lose some information about edges of size greater than two. Sometimes losing this information does not affect our ability to answer questions of interest, but in other cases it has a profound impact. In particular, an important scenario when hypergraph-based approach can be preferred, is when a large hyperedge connecting some vertices is a strong indicator that they all belong to the same community. Such situations occur often in practice. Let us briefly discuss two simple examples. First consider e-mails as hyperedges of a hypergraph whose vertices are e-mail addresses. Multiple addresses in an e-mail (group e-mails) most likely are not sent to random people, but rather to some community of common interests. As a second example consider a platform like GitHub, where vertices are users and hyperedges are repositories linking users that committed to them. Again, if a group of users commits to the same repository it is most likely a strong indicator that they form some community. In both cases, as indicated above, replacing a hyperedge by a clique would lose valuable information.

The paper is organized as follows. In Section 2, we review the Chung-Lu model for graphs and its link to the modularity function. We then propose a generalization of the Chung-Lu model for hypergraphs, as well as a hypergraph modularity function. In Section 3, we provide the framework to develop algorithms using our hypergraph modularity function. We propose an hypergraph partitioning algorithm and a few illustrative examples in Section 4. This is a new measure we are proposing, and there is plenty of future work to do, which we summarize in Section 5. Additionally, we made the source codes available on-line [19] allowing to reproduce the analyses presented in the paper.

## 2 Hypergraph modularity

In this section, we recall the definition of modularity function for graphs, and we propose its generalization for hypergraphs. Throughout the paper we will use *n* for the number of vertices. We will use (Xk) for the set consisting of all *k*-element subsets of *X*. Finally, [*n*] ≔ {1, …, *n*}.

### 2.1 Chung-Lu model for graphs

Let *G* = (*V*, *E*) be a graph, where *V* = {*v*_1_, …, *v*_*n*_} are the vertices, the edges *E* are multisets of *V* of cardinality 2 (loops allowed), and *deg*_*G*_(*v*) is the degree of *v* in *G* (with a loop at *v* contributing 2 to the degree of *v*). For *A* ⊆ *V*, let the *volume* of *A* be *vol*_*G*_(*A*) = ∑_*v*∈*A*_ *deg*_*G*_(*v*); in particular *vol*_*G*_(*V*) = ∑_*v*∈*V*_ *deg*_*G*_(*v*) = 2|*E*|. We will omit the subscript *G* when the context is clear.

We define G(G) to be the probability distribution on graphs on the vertex set *V* following the well-known Chung-Lu model [20–23]. In this model, each set *e* = {*v*_*i*_, *v*_*j*_}, *v*_*i*_, *v*_*j*_ ∈ *V*, is independently sampled as an edge with probability given by:

$$\begin{array}{c} P\left(v_{i},v_{j}\right)=\{\begin{array}{cc} \frac{deg\left(v_{i}\right)deg\left(v_{j}\right)}{2|E|}, & i\neq j\\ \frac{deg^{2}\left(v_{i}\right)}{4|E|}, & i=j. \end{array} \end{array}$$

(Let us mention about one technical assumption. Note that it might happen that *P*(*v*_*i*_, *v*_*j*_) is greater than one and so it should really be regarded as the expected number of edges between *i* and *j*; for example, as suggested in [24], one can introduce a Poisson-distributed number of edges with mean *P*(*v*_*i*_, *v*_*j*_) between each pair of vertices *i*, *j*. However, since typically the maximum degree Δ satisfies Δ^2^ ≤ 2|*E*| it rarely creates a problem and so we may assume that *P*(*v*_*i*_, *v*_*j*_) ≤ 1 for all pairs.)

This model is a function of the degree sequence of *G*. One desired property of this random model is that it yields a distribution that preserves the expected degree for each vertex, namely: for any *i* ∈ [*n*],

$$\begin{array}{ccc} E_{G^{\prime }∼G\left(G\right)}\left[deg_{G^{\prime }}\left(v_{i}\right)\right] & = & \sum_{j\in [n]\backslash \{i\}}\frac{deg\left(v_{i}\right)deg\left(v_{j}\right)}{2|E|}+2\cdot \frac{deg^{2}\left(v_{i}\right)}{4|E|}\\ & = & \frac{deg(v_{i})}{2|E|}\sum_{j\in [n]}deg\left(v_{j}\right)\;\;=\;\;deg\left(v_{i}\right), \end{array}$$

where all degrees are with respect to graph *G*. This model will be useful to understand the graph modularity definition and its generalization to hypergraphs.

### 2.2 Review of graph modularity

The definition of modularity for graphs was first introduced by Newman and Girvan in [8]. Despite some known issues with this function such as the “resolution limit” reported in [25], many popular algorithms for partitioning large graph data sets use it [26–28]. It was also recently studied for some models of complex networks [29–32]. The modularity function favours partitions in which a large proportion of the edges fall entirely within the parts (note that throughout the paper, we use the term “part” and “partition” that are more common in mathematical literature; in the information sciences the equivalent term is “cluster”) and biases against having too few or too unequally sized parts.

For a graph *G* = (*V*, *E*) and a given partition **A** = {*A*_1_, …, *A*_*k*_} of *V*, the modularity function is defined as follows:

$$\begin{array}{ccc} q_{G}\left(A\right) & = & \frac{1}{\left|E\right|}\sum_{A_{i}\in A}(e_{G}\left(A_{i}\right)-E_{G^{\prime }∼G\left(G\right)}\left[e_{G^{\prime }}\left(A_{i}\right)\right])\\ & = & \sum_{A_{i}\in A}\frac{e_{G}\left(A_{i}\right)}{\left|E\right|}-\sum_{A_{i}\in A}\frac{E_{G^{\prime }∼G\left(G\right)}\left[e_{G^{\prime }}\left(A_{i}\right)\right]}{\left|E\right|}, \end{array}$$ (1)

where *e*_*G*_(*A*_*i*_) = |{{*v*_*j*_, *v*_*k*_} ∈ *E*: *v*_*j*_, *v*_*k*_ ∈ *A*_*i*_}| is the number of edges in the subgraph of *G* induced by the set *A*_*i*_. The modularity measures the deviation of the number of edges of *G* that lie inside parts (clusters) of **A** from the corresponding expected value based on the Chung-Lu distribution G(G). The expected value for part *A*_*i*_ is

EG′∼G(G)[eG′(Ai)]=∑{vj,vk}∈(Ai2)deg(vj)deg(vk)2|E|+∑vj∈Aideg2(vj)4|E|=14|E|(∑vj∈Aideg(vj))2=(vol(Ai))24|E|.

The first term in (1), $\sum_{A_{i}\in A}e_{G}(A_{i})/|E|$, is called the *edge contribution*, whereas the second one, $\sum_{A_{i}\in A}{\left(vol(A_{i})\right)}^{2}/4|E{|}^{2}$, is called the *degree tax*. It is easy to see that *q*_*G*_(**A**) ≤ 1. Also, if **A** = {*V*}, then *q*_*G*_(**A**) = 0, and if **A** = {{*v*_1_}, …, {*v*_*n*_}}, then $q_{G}\left(A\right)=-\frac{\sum deg{\left(v\right)}^{2}}{{4|E|}^{2}}<0$.

The maximum *modularity* q*(*G*) is defined as the maximum of *q*_*G*_(**A**) over all possible partitions **A** of *V*; that is, q*(*G*) = max_**A**_ *q*_*G*_(**A**). In order to maximize *q*_*G*_(**A**) one wants to find a partition with large edge contribution subject to small degree tax. If q*(*G*) approaches 1 (which is the trivial upper bound), we observe a strong community structure; conversely, if q*(*G*) is close to zero (which is the trivial lower bound), there is no community structure. The definition in (1) can be generalized to weighted edges by replacing edge counts with sums of edge weights.

### 2.3 Generalization of the Chung-Lu model to hypergraphs

Consider a hypergraph *H* = (*V*, *E*) with *V* = {*v*_1_, …, *v*_*n*_}, where hyperedges *e* ∈ *E* are subsets of *V* of cardinality greater than one. Since we are concerned with not necessarily simple hypergraphs, hyperedges are multisets. Such hyperedges can be described using distincts sets of pairs *e* = {(*v*, *m*_*e*_(*v*)): *v* ∈ *V*} where $m_{e}\left(v\right)\in N\cup \left\{0\right\}$ is the multiplicity of the vertex *v* in *e* (including zero which indicates that *v* is not present in *e*). Then |*e*| = ∑_*v*_ *m*_*e*_(*v*) is the *size* of hyperedge *e* and the *degree of a vertex v* in *H* is defined as *deg*_*H*_(*v*) = ∑_*e*∈*E*_ *m*_*e*_(*v*). When the reference to the hyperedge is clear from the context, we simply use *m*_*i*_ to denote *m*_*e*_(*v*_*i*_).

A hypergraph is said to be *d*-*uniform* if all its hyperedges have size *d*. In particular, a 2-uniform hypergraph is simply a graph. All hypergraphs *H* can be expressed as the disjoint union of *d*-uniform hypergraphs *H* = ⋃ *H*_*d*_, where *H*_*d*_ = (*V*, *E*_*d*_), *E*_*d*_ ⊆ *E* are all hyperedges of size *d*, and $deg_{H_{d}}\left(v\right)$ is the *d*-degree of vertex *v*. As for graphs, the volume of a vertex subset *A* ⊆ *V* is *vol*_*H*_(*A*) = ∑_*v*∈*A*_ *deg*_*H*_(*v*).

Similarly to what we did for graphs, we define a random model on hypergraphs, H(H), where the expected degrees of all vertices are the corresponding degrees in *H*. To simplify the notation, we omit the explicit references to *H* in the remaining of this section; in particular, *deg*(*v*) denotes *deg*_*H*_(*v*), H denotes H(H), *E*_*d*_ denotes the edges of *H* of size *d*. Moreover, we use *E*′ to denote the edge set of *H*′.

Let *F*_*d*_ be the family of multisets of size *d*; that is,

$$\begin{array}{c} F_{d}≔\{\left\{\left(v_{i},m_{i}\right):1\leq i\leq n\right\}:\sum_{i=1}^{n}m_{i}=d\}. \end{array}$$

The hypergraphs in the random model are generated via independent random experiments. For each *d* such that |*E*_*d*_| > 0, the probability of generating the edge *e* ∈ *F*_*d*_ is given by:

PH(e)=|Ed|·(dm1,…,mn)∏i=1n(deg(vi)vol(V))mi. (2)

(Recall that *m*_*i*_ = *m*_*e*_(*v*_*i*_).) Let $\left(X_{1}^{\left(d\right)},\ldots ,X_{n}^{\left(d\right)}\right)$ be the random vector following a multinomial distribution with parameters $d,p_{H}\left(1\right),\ldots ,p_{H}\left(n\right)$, where $p_{H}\left(i\right)=deg\left(v_{i}\right)/vol\left(V\right)$ and $\sum_{i\in [n]}p_{H}\left(i\right)=1$; that is,

sH(e)≔P((X1(d),…,Xn(d))=(m1,…mn))=(dm1,…,mn)∏i=1n(pH(i))mi.

Note that this is the expression found in (2); that is, $P_{H}\left(e\right)=\left|E_{d}\right|\cdot s_{H}\left(e\right)$. As a result, alternatively one can think about the following auxiliary process. Select a random multiset consisting of *d* vertices (counting possible repetitions); in *d* independent rounds, vertex *v*_*i*_ is selected with probability $p_{H}\left(i\right)$. Repeat this experiment |*E*_*d*_| times and use the expected number of times edge *e* occurred in this process for the value of $P_{H}\left(e\right)$. An immediate consequence of this coupling between the two processes is that the expected number of edges of size *d* is |*E*_*d*_|. Finally, as with the graph Chung-Lu model, if $P_{H}\left(e\right)>1$, then it should be regarded as the expectation and multi-hypergraph should be considered instead. However, as before, from practical point of view it is safe to assume that all $P_{H}\left(e\right)\leq 1$.

In order to compute the expected *d*-degree of a vertex *v*_*i*_ ∈ *V*, note that

$$\begin{array}{c} deg_{H_{d}^{\prime }}\left(v_{i}\right)=\sum_{e\in F_{d}}m_{e}\left(v_{i}\right)\cdot I_{\left\{e\in E^{\prime }\right\}}, \end{array}$$

where $I_{\left\{\right\}}$ is the indicator random variable. Hence, using the linearity of expectation, then splitting the sum into *d* + 1 partial sums for different multiplicities of *v*_*i*_, we get:

$$\begin{array}{c} \begin{array}{ll} E_{H^{\prime }\sim H}(deg_{{H^{\prime }}_{d}}(v_{i})) & =\sum_{e\in F_{d}}m_{e}(v_{i})\cdot P_{H}(e)=|E_{d}|\sum_{e\in F_{d}}m_{e}(v_{i})\cdot s_{H}(e)\\ & =|E_{d}|\sum_{m=0}^{d}m\sum_{e\in F_{d};m_{e}(v_{i})=m}s_{H}(e)\\ & =|E_{d}|\sum_{m=0}^{d}m\cdot \mathbb{P}(X_{i}^{\left(d\right)}=m)\\ & =|E_{d}|\sum_{m=0}^{d}m\cdot \left(\begin{array}{c} d\\ m \end{array}\right){\left(p_{H}(i)\right)}^{m}{\left(1-p_{H}(i)\right)}^{d-m}\\ & =|E_{d}|\cdot d\cdot p_{H}(i). \end{array} \end{array}$$

The second last equality follows from the fact that we obtained the expected value of a random variable with binomial distribution. One can compute the expected degree as follows:

$$\begin{array}{c} E_{H^{\prime }∼H}\left[deg_{H^{\prime }}\left(v_{i}\right)\right]=\sum_{d\geq 2}\frac{d\cdot |E_{d}|\cdot deg\left(v_{i}\right)}{vol(V)}=deg\left(v_{i}\right), \end{array}$$

since *vol*(*V*) = ∑_*d*≥2_ *d* ⋅ |*E*_*d*_|.

We will use the generalization of the Chung-Lu model to hypergraphs as a null model allowing us to define hypergraph modularity.

### 2.4 Hypergraph modularities

Consider a hypergraph *H* = (*V*, *E*) and **A** = {*A*_1_, …, *A*_*k*_}, a partition of *V*. For edges of size greater than 2, several definitions can be used to quantify the edge contribution given **A**, such as:

(a) all vertices of an edge have to belong to one of the parts (clusters) to contribute; this is a *strict* definition that we focus on in this paper;
(b) the *majority* of vertices of an edge belong to one of the parts;
(c) at least 2 vertices of an edge belong to the same part; this is implicitly used when we replace a hypergraph with its 2-section graph representation.

We see that the choice of hypergraph modularity function is not unique; in fact, it depends on how strongly we believe that a hyperedge is an indicator that vertices belonging to it fall into one community. More importantly, one needs to decide how often vertices in one community “blend” together with vertices from other community; that is, how hermetic the community is. In particular, option (c) is the *softest* one and leads to standard 2-section graph modularity. In order to illustrate how one can obtain formulas for a given variant, we concentrate on the second extreme, option (a), that we call *strict*. However, it is easy to repeat calculations for other variants. We will skip details but differences will be highlighted and the final formula for option (b) will be presented next. Comparison of various variants will be done in the forthcoming paper.

#### 2.4.1 Strict hypergraph modularity

In this case, the definition of edge contribution for *A*_*i*_ ⊆ *V* is:

$$\begin{array}{c} e_{H}\left(A_{i}\right)=\left|\left\{e\in E;\;e\subseteq A_{i}\right\}\right|. \end{array}$$ (3)

The strict modularity of **A** on *H* is then defined as a natural extension of standard modularity in the following way:

$$\begin{array}{c} q_{H}\left(A\right)=\frac{1}{\left|E\right|}\sum_{A_{i}\in A}(e_{H}\left(A_{i}\right)-E_{H^{\prime }∼H}\left[e_{H^{\prime }}\left(A_{i}\right)\right]). \end{array}$$ (4)

Consider any *A* ⊆ *V*. We want to compute the expected edge contribution of *A* over H. Let *F*_*d*_(*A*) ⊆ *F*_*d*_ be the family of multisets of size *d* with all members in *A*; that is,

$$\begin{array}{c} F_{d}\left(A\right)≔\{\left\{\left(v_{i},m_{i}\right):1\leq i\leq n\right\}:\sum_{i=1}^{n}m_{i}=\sum_{i:v_{i}\in A}m_{i}=d\}. \end{array}$$

First, note that

$$\begin{array}{ccc} E_{H^{\prime }∼H}\left[e_{H^{\prime }}\left(A\right)\right] & = & \sum_{d\geq 2}\sum_{e\in F_{d}\left(A\right)}P_{H}\left(e\right)=\sum_{d\geq 2}\left|E_{d}\right|\sum_{e\in F_{d}\left(A\right)}s_{H}\left(e\right)\\ & = & \sum_{d\geq 2}|E_{d}|\cdot P(\sum_{i;v_{i}\in A}X_{i}^{\left(d\right)}=d)=\sum_{d\geq 2}\left|E_{d}\right|\cdot {\left(p_{H}\left(A\right)\right)}^{d} \end{array}$$ (5)

where $p_{H}\left(A\right)=\sum_{i;v_{i}\in A}p_{H}\left(i\right)$, therefore $p_{H}\left(A\right)=vol\left(A\right)/vol\left(V\right)$, so

$$\begin{array}{cc} E_{H^{\prime }∼H}\left[e_{H^{\prime }}\left(A\right)\right] & =\sum_{d\geq 2}\left|E_{d}\right|\cdot {\left(vol\left(A\right)/vol\left(V\right)\right)}^{d}. \end{array}$$ (6)

Putting (6) properly into (4), we get the strict modularity function of a hypergraph partition:

$$\begin{array}{ccc} q_{H}\left(A\right) & = & \frac{1}{\left|E\right|}(\sum_{A_{i}\in A}e\left(A_{i}\right)-\sum_{d\geq 2}\left|E_{d}\right|\sum_{A_{i}\in A}(\frac{vol(A_{i})}{vol(V)}{)}^{d}). \end{array}$$ (7)

Just as for graphs, the corresponding *modularity* $q_{H}^{*}$ is defined as the maximum of *q*_*H*_(**A**) over all possible partitions **A** of *V*.

#### 2.4.2 Generalizations

As with graphs, one can easily generalize the modularity function to allow for weighted hyperedges. As we already mentioned, we focused on the strict definition of modularity but it is straightforward to adjust the degree tax to many natural definitions of edge contribution. In particular, for the majority definition (see option (b) at the beginning of this section), one can simply replace $P(\sum_{i;v_{i}\in A}X_{i}^{\left(d\right)}=d)$ with $P(\sum_{i;v_{i}\in A}X_{i}^{\left(d\right)}>d/2)$ in (5), and so (*vol*(*A*)/*vol*(*V*))^*d*^ in (6) (that is equivalent to P(Bin(d,vol(A)/vol(V))=d) becomes P(Bin(d,vol(A)/vol(V))>d/2). The majority modularity function of a hypergraph partition is then:

$$\begin{array}{ccc} q_{H}\left(A\right) & = & \frac{1}{\left|E\right|}(\sum_{A_{i}\in A}e\left(A_{i}\right)-\sum_{d\geq 2}\left|E_{d}\right|\sum_{A_{i}\in A}P(\text{Bin}(d,\frac{vol(A_{i})}{vol(V)})>d/2)). \end{array}$$

We can also consider the modularity independently over hyperedges of different sizes. Decomposing *H* into *d*-uniform hypergraphs *H*_*d*_, we get the following degree-independent modularity function:

$$\begin{array}{c} q_{H}^{DI}\left(A\right)=\sum_{d\geq 2}\frac{|E_{d}|}{\left|E\right|}q_{H_{d}}\left(A\right). \end{array}$$

This corresponds to (7) replacing the volumes computed over *H* with volumes computed over *H*_*d*_ for each *d* where |*E*_*d*_| > 0.

## 3 Searching the solution space

In this section, we show that the solution that maximizes (7) lies in a subset of P(V) of size at most 2^|*E*|^ avoiding the search of the full set P(V). Of course, it is still not feasible to check all subsets of the edge set but observations made in this section will be useful for designing efficient heuristic algorithms. For example, in this paper we already used a simple CNM-like algorithm (see Algorithm 1) in which edges overlapping with at least two parts clusters) have to be considered. Using the results from this section, we can significantly reduce the search space by ignoring edges that fall into one part. More sophisticated (and, hopefully, much faster) algorithms utilizing properties investigated in this section will be developed in the forthcoming paper.

Let S(H) denote the set of all sub-hypergraphs of *H* = (*V*, *E*) on the vertex set *V*: $S\left(H\right)=\{H^{\prime }=\left(V,E^{\prime }\right)\;|\;E^{\prime }\subseteq E\}$. We use |*H*′| to denote |*E*′|, the number of edges in *H*′. Moreover, let p:S(H)→P(V) denote the function that sends a sub-hypergraph of *H* to the partition its connected components induce on *V*. We define a relation on S(H):

$$\begin{array}{c} H_{1}\equiv_{p}H_{2}\Leftrightarrow p\left(H_{1}\right)=p\left(H_{2}\right) \end{array}$$

that puts two sub-hypergraphs in relation if they have identical connected components. Since ≡_*p*_ is an equivalence relation (based on equality), we can define the quotient set $S\left(H\right){/}_{\equiv_{p}}$. This quotient set contains equivalence classes that are in bijection with the set of all different vertex partitions that can be induced by the union of elements of *E*. Its cardinality depends on *E* but is at most 2^|*E*|^; however, it is typically much smaller than this trivial upper bound.

Now, let us define the *canonical representative mapping* which identifies a natural representative member for each equivalence class. The canonical representative mapping $f:S\left(H\right){/}_{\equiv_{p}}\to S\left(H\right)$ maps an equivalence class to the largest member of this class: *f*([*H*′]) = H* where H* ∈ [*H*′] and |H*| ≥ |*H*″| for all *H*″ ∈ [*H*′]. This function is well-defined; indeed, if *H*_1_, *H*_2_ ∈ [*H*′], then the union of *H*_1_ and *H*_2_ is also in [*H*′] and so it is impossible that two members have the largest size. Its outcome is the subgraph H* = (*V*, E*) whose edge set is the union of edges of all members of the equivalence class. The following lemma explains why the canonical representative is *natural* with respect to the definition of strict modularity. As this observation follows easily from definitions, the proof is omitted.

**Lemma 3.1**. *Let H* = (*V*, *E*) *be a hypergraph and* **A** = {*A*_1_,…, *A*_k_} *be any partition of V. If there exists* $H^{\prime }\in S\left(H\right)$ *such that* **A** = *p*(*H*′), *then the edge contribution of the strict modularity of* **A** is $\frac{|E^{*}|}{\left|E\right|}$, where E* *is the edge set of the canonical representative of* [*H*′].

The set of canonical representatives, the image of *f*, is a subset of S(H). We denote this set by $S^{*}\left(H\right)$ and the image of *p* restricted to $S^{*}\left(H\right)$ by $P^{*}\left(V\right)$.

The next Lemma shows how the degree tax behaves on partition refinement.

**Lemma 3.2**. *Let H* = (*V*, *E*) *be a hypergraph and* **A** *be any partition of V*. *If* **B** *is a refinement of* **A**, *then the degree tax of* **A** *is larger than or equal to the degree tax of* **B** *and it is equal if and only if* **A** = **B**.

*Proof*. Let **A** = {*A*_1_,…, *A*_k_}. Since **B** is a refinement of **A**, for each part (cluster) of **A**, *A*_*i*_, there exists **B**_*i*_, a subset of parts of **B**, such that $A_{i}={⋃}_{B\in B_{i}}B$ and **B** = ⋃_*i*_ **B**_*i*_. Hence, for each *A*_*i*_ and for each *d*, we have that $vol_{d}\left(A_{i}\right)=\sum_{B\in B_{i}}vol_{d}\left(B\right)$ and so

$$\begin{array}{c} vol_{d}{\left(A_{i}\right)}^{d}=(\sum_{B\in B_{i}}vol_{d}\left(B\right){)}^{d}\geq \sum_{B\in B_{i}}vol_{d}{\left(B\right)}^{d}. \end{array}$$

The equality holds if and only if |**B**_*i*_| = 1 for all *i*. The result follows.

The next result, the main result of this section, shows that one can restrict the search space to canonical representatives from $S^{*}\left(H\right)$.

**Theorem 3.3**. *Let H* = (*V*, *E*) *be a hypergraph. If* A∈P(V) *maximizes the strict modularity function q*_*H*_(⋅), *then* $A\in P^{*}\left(V\right)$.

*Proof*. Assume that **A** = {*A*_1_,…, *A*_k_} maximizes the strict modularity function *q*_*H*_(⋅). We will show that there exists $H^{*}=\left(V,E^{*}\right)\in S^{*}\left(H\right)$ such that *q*_*H*_(*p*(H*)) ≥ *q*_*H*_(**A**). Let E* = {*e* ∈ *E*: *e* ⊆ *A*_*i*_ for some *i*}. By construction of H*, the (strict) edge contribution of partitions **A** and *p*(H*) are identical. Again, from construction, note that the partition *p*(H*) is a refinement of **A**. Hence, the previous Lemma states that the degree tax of **A** is larger than or equal to the degree tax of *p*(H*). With equal edge contribution, this means that *q*_*H*_(*p*(H*)) ≥ *q*_*H*_(**A**). Since **A** is an optimal solution, the equality must hold which is only possible if **A** = *p*(H*).

## 4 Examples

In this section, we first illustrate the correlation between our hypergraph modularity and the Hcut measure, which counts the number of edges touching more than one part (cluster). We then propose an algorithm for hypergraph partitioning based on our hypergraph modularity function, which we apply to a real dataset. However, let us stress again that the aim of this paper is to introduce a generalization of the modularity to hypergraphs, not to introduce new algorithms to actually find good partitions. We currently work on designing and testing algorithms for the hypergraph counterpart and, after that, we plan to do extensive experiments. The results will be included in the forthcoming paper.

### 4.1 Synthetic hypergraphs

We generate hyperedges following the process described as *line clustering* in section 5.1 of [33]. We first randomly generate points from 3 lines in the range [−.5, .5]^2^, 30 points per line, perturbed with Gaussian noise *N*(0, *σ*^2^) where *σ* = 0.01. Each line cuts the origin, and the respective slopes are -1, 0.02 and 0.8. We also generate 60 outlying points chosen at random over the same range, for a total of 150 points.

We build hyperedges of size 3 (3-edges) by sampling sets of 3 points {*i*, *j*, *k*} for which $\text{exp}(-d{\left(i,j,k\right)}^{2}/\sigma_{d}^{2})>0.999$, where *d*() is the mean distance of the points to their best fitting line, and *σ*_*d*_ = 0.02. This amounts to selecting 3-edges consisting of sets of 3 well aligned points. We do the same with sets of 4 points to generate the 4-edges.

The hyperedges can either consist of points all coming from the same line (which we call “signal”) or not (which we call “noise”). We sample hyperedges so that the expected proportion of signal vs. noise is 2:1, and we consider 3 different regimes for the mix of edge sizes: (i) 75% 3-edges, (ii) 75% 4-edges or (iii) balanced between 3 and 4-edges. For the 3 regimes, we generate 100 hypergraphs and for each hypergraph, we apply the fast Louvain clustering algorithm (see [34]) on the weighted 2-section graph. In most cases, vertices coming from the same line are correctly put in the same part (cluster). The complete source code for this example along with instructions is available online [19].

**Fig 1:**
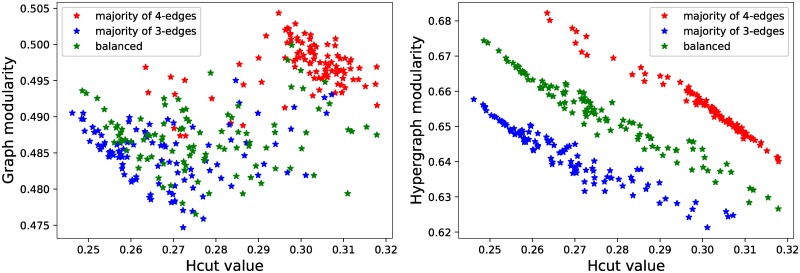
Modularity vs Hcut. Comparing graph and hypergraph modularity.

In the left plot of Fig 1, we plot the standard graph modularity vs. the Hcut value, which is simply the proportion of hyperedges that fall in two or more parts. The Louvain algorithm is not explicitly aiming at preserving the hyperedges, so we do not expect a high correlation between the two measures. In fact, fitting a regression line to the points from the balanced regime, we get a slope of 0.0061 with *R*^2^ value of 0.0008 (for majority of 4-edges the slope is 0.0768 and *R*^2^ 0.0734, while for majority of 3-edges the slope is 0.0061 and *R*^2^ 0.0004).

In the right plot of Fig 1, we do the same, this time comparing our hypergraph modularity with the Hcut values for the same partitions as in the left plot. The correlation here is very high. For the balanced regime, linear regression yields a slope of -0.6364 with *R*^2^ value of 0.9693 (for majority of 4-edges the slope is -0.7079 and *R*^2^ 0.9696, while for majority of 3-edges the slope is -0.7079 and *R*^2^ 0.9696). This is an illustration of the fact that when we measure our proposed hypergraph modularity for different partitions, we are favouring keeping hyperedges in the same parts (clusters).

### 4.2 Estimating the modularity

While we can compute the modularity for very small hypergraphs by exhausting over all possible partitions, this is generally not a viable option. We propose a generalization to hypergraphs of the CNM algorithm for graph partitioning [26]. In the CNM algorithm, we start with every vertex in its own part. At each step, we merge the two parts (clusters) that yield the largest increase in modularity, and we repeat until no such move exists. The algorithm comes in two versions. In the full version at each step all hyperedges are searched and evaluated for merging. Since for larger hypergraphs this would be prohibitively numerically expensive, in the stochastic version at each step we evaluate just one randomly chosen hyperedge.

Our proposed algorithm for hypergraphs is presented in Algorithm 1. The idea is that we start with a partition where each node is in its own part. Then in each step, we loop through every hyperedge touching two or more parts, and we select (or we just randomly chose a single hyperedge) the one which, when we merge all the parts it touches, yields the best modularity, provided it is at least as high as the modularity from the previous step. We stop when no such edge exists. We use this algorithm in the next example.

**Algorithm 1**: **Simple CNM-like algorithm on a hypergraph** *H*

**Data**: hypergraph *H* = (*V*, *E*)

**Result**: **A**_*opt*_, a partition of *V* with modularity *q*_*opt*_

1 Initialize E* = *E* and **A**_*opt*_ the partition with all *v* ∈ *V* in its own part with *q*_*opt*_ the corresponding modularity;

2 **repeat**

3  **if** *(using simplified stochastic algorithm version)* **then**

4   e* = *rand*(E*) #randomly select an edge;

5   compute the partition *A*_e*_ obtained when merging all parts in **A**_*opt*_ touched by e*, and compute its modularity *q*_e*_;

6  **end**

7  **else**

8   **foreach** *e* ∈ E* **do**

9     compute the partition *A*_*e*_ obtained when merging all parts in **A**_*opt*_ touched by *e*, and compute its modularity *q*_*e*_;

10   **end**

11   select edge e* ∈ E* with highest *q*_e*_;

12  **end**

13  **if** *q*_e*_ ≥ *q*_*opt*_ **then**

14   *A*_*opt*_ = *A*_e*_ and *q*_*opt*_ = *q*_e*_;

15   update E*, the set of edges touching two or more parts in **A**_*opt*_;

16  **end**

17 **until** (*q*_e*_ < *q*_*opt*_) ***or*** E* = ∅ ***or*** *computational time budget exceeded*;

18 output: **A**_*opt*_ and *q*_*opt*_

We have implemented the simplified stochastic version of the algorithm in Julia and use it on the hypergraph generated with the forementioned regime (i) that has 75% edges of degree 3 and 25% edges of the degree 4. In our implementation of Algorithm 1, we choose hyperedges to consider at random order (that is, at each step one hyperedge is randomly selected). In order to validate the algorithm, we have run it 1920 times with 500 steps at each run. A single run on a hypergraph consisting of 150 vertices and 5094 hyperedges, using a modern CPU and a single thread, took around 7 seconds. Note that the computational complexity of the proposed algorithm is *O*(*n*) and hence it can be practically used for real-world hypergraphs.

**Fig 2:**
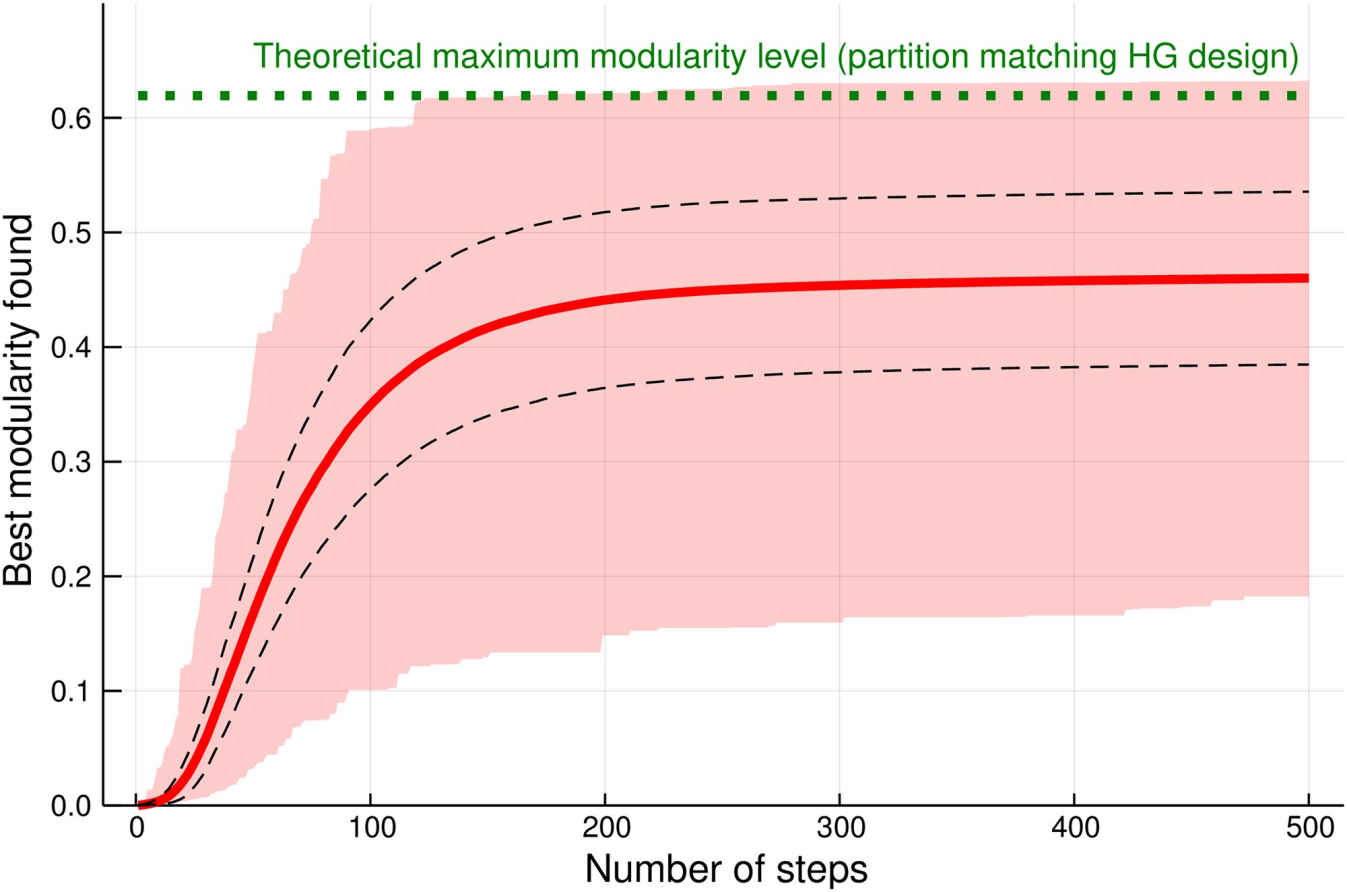
Performance of the Algorithm 1. The thick red line represents the average performance, the dashed line represent standard deviation for the performance and the wide pink area represents the smallest and the biggest performance found in all experiments.

The results presented in Fig 2 show that the heuristics presented in Algorithm 1 leads to practically reasonable and useful communities. Firstly, on average, the modularity level after 500 steps is around 75% on modularity for an “optimal” partition of the hypergraph (that is, the partition that uses the knowledge on how the hypergraph was generated). Secondly, this result can be improved by rerunning the Algorithm 1 several times (and this process can be parallelized in high performance computing environments). In particular, we have noticed that for 1.2% of all runs a partition having the optimal modularity (at least 0.6194 for the partition of the partition that matched the process of data generation described in the section 4.1 where we have three groups of points (30 points in group) sharing common hyperedges in each group and an additional 60 points) have been found. Please also note that a higher observed empirical value was observed in some cases—this arises due to the fact that the for last group of synthetic hypergraph having 60 vertices the hyperedges have been generated randomly.

### 4.3 DBLP hypergraph

The DBLP computer science bibliography database contains open bibliographic information on major computer science journals and proceedings. The DBLP database is operated jointly by University of Trier and Schloss Dagstuhl. The DBLP Paper data is available at http://dblp.uni-trier.de/xml/.

We consider a hypergraph of citations where each vertex represents an author and hyperedges are papers. In order to properly match author names across papers we enhance the data with information scraped from journal web pages. The DBLP database contains the doi.org identifier. We use this information to obtain the journal name and retrieve paper author data directly from journal—we update available author name data using ACM, IEEE Xplore, Springer and Elsevier/ScienceDirect databases. Since the same author names can be written differently we match author names of the paper across all three data sources. This can give good representation of author names for later matching. For the analysis, we only kept the (single) large connected component. We obtained a hypergraph with 1637 nodes, 865 edges of size 2, 470 of size 3, 152 or size 4 and 37 of size 5 to 7. The complete source code for this example along with instructions and data files is available online [19].

In Table 1, we show our results with the Louvain algorithm on the 2-section graph using modularity function *q*_*G*_(), as well as the results with our CNM algorithm on hypergraphs. Comparing the Louvain and CNM algorithms, we see that there is a tradeoff between *q*_*H*_ and *q*_*G*_ and moreover, the Hcut value is lower with the CNM algorithm. The increased number of parts with our algorithms is mainly due to the presence of singletons.

**Table 1:** Partitioning DBLP dataset.

algorithm	*q*_*H*_()	*q*_*G*_()	Hcut	#parts
Louvain	0.8613	0.8805	0.1181	40
CNM	0.8671	0.8456	0.0945	92

Another observation is that the actual partitions obtained with objective function *q*_*G*_() (Louvain) and *q*_*H*_() (CNM) are different. For the Louvain and CNM algorithms, we found values of 0.4355 for the adjusted Rand index (ARI), 0.4416 for its graph-aware counterpart (see [35]) and 0.684 for the adjusted mutual information, which are commonly used measures of comparisons for partitions. Similar partitions would show values close to 1. We used adjusted measures which are preferable to non-adjusted ones (such as NMI, the normalized mutual information) as they correct for random chance.

One of the difference lies in the number of edges of size 2, 3 and 4 that are cut with the different algorithms, as we see in Table 2. The algorithms based on *q*_*H*_() will tend to cut less of the larger edges, as compared to the Louvain algorithm, at expense of cutting more size-2 edges.

**Table 2:** Proportion of edges of size 2, 3 or 4 cut by the algorithms.

Algorithm	2-edges	3-edges	4-edges
Louvain	0.0382	0.1815	0.3158
CNM	0.0590	0.1277	0.1842

## 5 Conclusion

In this paper, we presented a generalization of the Chung-Lu model for hypergraphs, which we used to define a modularity function on hypergraphs. Interestingly, in hypergraph modularity case there is no one unique way to define modularity and we show that it depends on how strongly we think that a hyperedge indicates members of the same community. If the belief is soft this leads to standard 2-section graph modularity. However, if it is strong, a natural definition is strict hypergraph modularity, which we tested on numerical examples. We also proposed the in-between majority-based modularity function.

The objective of this paper is to develop a definition of hypergraph modularity. However, in order to show that this notion is numerically traceable, at least approximately, we provided the theoretical foundations for the development of algorithms using this modularity function that greatly reduce the solution search space.

A key natural question with any new measure is if it provides qualitatively different outcomes than existing ones. Therefore we have compared strict hypergraph modularity with a standard 2-section graph modularity. For this we proposed a simple heuristic algorithm. We illustrated the fact that in comparison to 2-section graph modularity (optimized using Louvain algorithm) optimization using strict modularity function tends to cut a smaller number of hyperedges. Therefore the proposed measure is potentially highly valuable in application scenarios, where a hyperedge is a strong indicator that vertices it contains belong to the same community.

Hypergraph modularity is a new measure, and there is still a lot of work that should be done. First of all, the development of good, efficient heuristic algorithms would allow to look at larger hypergraphs. Such algorithms would allow us to perform a study over hypergraphs with different edge size distributions, comparing the hypergraph modularity function with other definitions such as graph modularity over the 2-section representation of the hyperedges, and hypergraph modularity using the less strict majority rule.

Finally, let us mention that the method of modularity maximization (in its generalized form which incorporates a resolution parameter controlling the size of the communities discovered) is equivalent to another widely used methods of community detection in networks, the method of maximum likelihood applied to the special case of the stochastic block model known as the planted partition model, in which all communities in a network are assumed to have statistically similar properties [36] (see also [37]). It would be interesting (and potentially useful) to investigate if there is a natural counterpart of our hypergraph modularity measure.
